# Using network analysis to explore the association between eating disorders symptoms and aggressiveness in Bulimia nervosa

**DOI:** 10.1192/j.eurpsy.2024.1160

**Published:** 2024-08-27

**Authors:** R. Ceres, M. Battipaglia, N. Attianese, S. Donato, R. Cerra, G. D’Agostino, A. M. Monteleone, P. Monteleone, G. Cascino

**Affiliations:** ^1^Department of Mental, Physical Health and Preventive Medicine, University of Campania “Luigi Vanvitelli”, Naples; ^2^Department of Medicine, Surgery and Dentistry, University of Salerno “Scuola Medica Salernitana”, Salerno, Italy

## Abstract

**Introduction:**

Aggressive behaviors have been reported to be more frequent in people with eating disorders (ED), especially bulimia nervosa (BN). Network Analysis (NA) is particularly useful or examining the interactions among symptoms of comorbid conditions through the identification of “bridge symptoms,” defined as those symptoms playing a key role in the connection between two syndromic clusters.

**Objectives:**

The aim of the present study was to investigate the association of ED core symptoms and ED-related psychopathology with aggressiveness in a clinical sample of women with BN through NA.

**Methods:**

A NA was conducted, including ED symptoms and aggressiveness measures. The bridge function was implied to identify symptoms bridging ED symptoms and aggressiveness.

**Results:**

The most connected nodes among communities were asceticism and impulsivity from ED-related psychopathology, drive for thinness from ED- core psychopathology and guilt and suspicion from aggressiveness domain. In particular, drive for thinness connected ED-core community to verbal hostility, while impulsivity connected ED-related symptoms to guilt and suspicion of aggressiveness community.

**Conclusions:**

In conclusion the present study showed that in people with BN guilt is the specific negative emotion of the hostile dimensions that may be bidirectionally associated with ED symptoms.

**Disclosure of Interest:**

None Declared

